# Detection of carbapenem resistance among third-generation cephalosporin-resistant Enterobacterales from small-scale poultry farms in peri-urban Lusaka, Zambia

**DOI:** 10.1099/acmi.0.001108.v4

**Published:** 2026-01-28

**Authors:** Situmbeko J. Nasilele, Misheck Shawa, Harvey K. Kamboyi, Bruno S. J. Phiri, Tapiwa Lundu, Mike Nundwe, Angela Lungu, Ladslav Moonga, Shohei Ogata, Masahiro Kajihara, Hirofumi Sawa, Yasuhiko Suzuki, Hideaki Higashi, Ntombi B. Mudenda, Mudenda B. Hang’ombe, Kaampwe Muzandu

**Affiliations:** 1 Biomedical Sciences Department, School of Veterinary Medicine, University of Zambia, Lusaka, 10101, Zambia; 2 Hokudai Center for Zoonosis Control in Zambia, University of Zambia, Lusaka, 10101, Zambia; 3 Division of International Research Promotion, International Institute for Zoonosis Control, Hokkaido University, N20 W10, Kita-ku, Sapporo 001-0020, Japan; 4 Department of Clinical Medicine, School of Medicine, Eden University, Lusaka, Zambia; 5 Department of Para-clinical Studies, School of Veterinary Medicine, University of Zambia, Lusaka, 10101, Zambia; 6 Institute of Basic and Biomedical Sciences, Levy Mwanawasa Medical University, Lusaka 10101, Zambia; 7 Department of Disease Control, School of Veterinary Medicine, University of Zambia, Lusaka, 10101, Zambia; 8 Institute for Vaccine Research and Development (HU-IVReD), Hokkaido University, N21 W11, Kita-ku, Sapporo 001-0020, Japan; 9 Division of Bioresources, International Institute for Zoonosis Control, Hokkaido University, Kita-ku, Sapporo, Japan; 10 Division of International Research Promotion, International Institute for Zoonosis Control, Hokkaido University, N20 W10, Kita-ku, Sapporo 001-0020, Japan; 11 Department of Clinical Studies, School of Veterinary Medicine, University of Zambia, Lusaka, 10101, Zambia; 12 Division of Research and Innovation, Copperbelt University, Jambo Drive, Riverside, Kitwe, 10101, Zambia; 13 Africa Centre of Excellence for Infectious Diseases of Humans and Animals, School of Veterinary Medicine, University of Zambia, Lusaka, Zambia

**Keywords:** antimicrobial resistance (AMR), carbapenem resistance, Enterobacterales, poultry, third-generation cephalosporin (3GC) resistance, Zambia

## Abstract

**Background.** Carbapenem and third-generation cephalosporin (3GC) resistance among Gram-negative bacteria poses a serious threat to human and animal health. This study aimed to identify and characterize carbapenem- and 3GC-resistant Enterobacterales isolated from poultry in Lusaka Province, Zambia.

**Methods.** Ninety pooled cloacal samples were collected from market-ready broiler chickens in the Chongwe and Chilanga districts of Lusaka Province. The isolates were screened for 3GC and carbapenem resistance using the disc diffusion and broth microdilution methods. PCR and Sanger sequencing were performed for species identification and detection of β-lactamase-encoding (*bla*) genes, including *bla*_CTX-M_, *bla*_TEM_, *bla*_OXA-1_ and *bla*_SHV_. Hierarchical clustering was used to assess phenotypic and genotypic relationships.

**Results.** A total of 83 3GC-resistant Gram-negative isolates were recovered, of which 12% were also carbapenem resistant. *Escherichia coli* was the most prevalent species, followed by *Klebsiella pneumoniae* and *Enterobacter* spp., then *Pseudomonas aeruginosa,* other *Pseudomonas* spp., *Acinetobacter baumannii*, *Citrobacter freundii* and *Aeromonas caviae*. Multidrug resistance occurred in 84.3% of the isolates, with the highest resistance to ampicillin, tetracycline and co-trimoxazole. Overall, 80.7% of the isolates harboured at least one of the four tested *bla* genes, with *bla*_CTX-M_ and *bla*_TEM_ being the most common. Hierarchical clustering revealed that isolates from both districts shared similar phenotypic and genotypic resistance patterns.

**Conclusions.** The presence of multidrug- and carbapenem-resistant Enterobacterales from poultry highlights the emergence of carbapenem resistance in Zambia’s food production sector. The detection of imipenem-resistant isolates indicates the potential for transmission of resistance genes between animals and humans. These findings underscore the need for prudent antimicrobial use, strengthened stewardship and a One Health surveillance approach to contain the spread of carbapenem resistance genes.

**Impact Statement**

This study highlights the burden of carbapenem resistance and the high occurrence of drug-resistant Enterobacterales carrying multiple β-lactamase genes in poultry from small-scale farms in Zambia. This highlights a serious public health threat posed by the growing risk of antimicrobial resistance transfer from food animals to humans, underscoring the urgency of coordinated action to promote prudent antibiotic use.

## Data Summary

All data and materials from this study are available within the manuscript, its supplementary files or public repositories. Antimicrobial susceptibility data are presented in the main text and Table S1. Sanger sequencing data are deposited in GenBank (accession: PX232362–PX232368; submission: SUB15567876). Data were imported into R v4.4.1 and manipulated using the dplyr package [1], before being visualized with ggplot2 [2]. We used hierarchical clustering (ComplexHeatmap package) [3] to group isolates by antibiotic resistance profiles and *bla* genes. Additional figures were created in Excel and GraphPad Prism [4], while statistical comparisons of categorical data employed chi-square or Fisher’s exact tests from the epiR package [5].

## Introduction

The emergence and spread of antimicrobial resistance (AMR) among bacterial pathogens pose a significant global public health threat, particularly in low- and middle-income countries, where surveillance and regulatory frameworks remain limited [6,8]. Misuse of antibiotics in both the human and animal health sectors has accelerated the evolution of multidrug-resistant bacterial strains. Globally, antimicrobial use in poultry farming is widespread. In Zambia, tetracyclines, sulphonamides and penicillins are among the most commonly used antimicrobials [9,10]. Similarly, penicillins and cephalosporins are frequently prescribed in human medicine [11,12]. Carbapenems are often reserved for treating infections caused by extended-spectrum β-lactamase (ESBL)-producing Enterobacterales, emphasizing the clinical significance of emerging carbapenem resistance [13,16].

Over the past few decades, carbapenem-resistant Gram-negative bacteria (CR-GNB), including Enterobacterales and non-fermenters such as *Pseudomonas* and *Acinetobacter*, have emerged as a global public health concern, driven by diverse AMR mechanisms [17,19]. In Zambia, the human health sector has observed a rise in bacterial isolates resistant to carbapenems and third-generation cephalosporins (3GCs) [11]. Although carbapenems are not used in poultry production, recent Zambian studies have identified CR-GNB in poultry, but the isolation frequencies remain very low [20,22]. In contrast, 3GC resistance among Enterobacterales is a major concern in Zambia’s poultry industry [20,21, 23, 24], often linked to plasmid-mediated β-lactamase genes that co-harbour multiple AMR determinants, resulting in multidrug resistance (MDR) [25,27].

Despite growing reports of 3GC resistance and MDR among Enterobacterales in the Zambian poultry sector, there remains a paucity of information on CR-GNB. AMR surveillance has largely focused on clinical settings, thereby limiting understanding of potential animal reservoirs that contribute to the overall AMR burden. While a few studies have included AMR patterns in poultry, most have primarily documented ESBL production among 3GC-resistant Enterobacterales [9]. Despite our previous report in Copperbelt Province [28], there is limited information on carbapenem resistance mechanisms and the molecular basis of CR-GNB in the poultry environment in Zambia. The current study provides the first focused investigation into carbapenem resistance among Gram-negative bacteria from poultry farms in Lusaka, Zambia, thereby expanding the geographic scope. The novelty of this study is relevant in guiding AMR surveillance and intervention strategies in Zambia.

## Methods

### Study area and sampling

The study targeted small-scale poultry farmers in Lusaka Province, Zambia, with a total human population of 3,079,964 [29]. Of the 46,927 small-scale broiler farms in the country, Lusaka Province accounts for 9,397 (20%), second only to Copperbelt Province (12,521, 26.7%) [30]. However, Lusaka has the highest concentration of broiler production areas per square kilometre and the most commercial poultry abattoirs, underscoring its role as the leading consumer of poultry and poultry products in Zambia [31,32].

As part of the Zambia National AMR Surveillance, a cross-sectional study was conducted on 45 randomly selected small-scale farms in Chongwe (*n*=35) and Chilanga (*n*=10) districts in Lusaka Province between August and December 2024. Chongwe district was allocated more samples due to a higher human population [33] and a larger number of eligible small-scale poultry farms. Farms were included if they housed 100–1,000 market-ready chickens.

From each farm, one to three cloacal swabs were collected from market-ready broiler chickens. Next, the chickens were slaughtered in accordance with strict hygiene protocols, and meat swabs were aseptically collected immediately after defeathering. One cloacal swab and one meat swab were independently collected per 100 chickens and pooled into a single sample per small-scale farm; thus, multiple cloacal and meat swabs were collected from farms with more than 100 chickens. Cloacal and meat swabs were independently preserved in Amies transport media (Mantacc, Miraclean Technology Co., Ltd., China) and placed on ice while being transported to the University of Zambia School of Veterinary Medicine laboratory for processing and analysis.

### Isolation of 3GC-resistant Enterobacterales

Pooled cloacal and meat swabs from each farm were separately pre-enriched in 9 ml of Buffered Peptone Water (BPW; HiMedia, Pvt. Ltd., India) and incubated at 37 °C for 18 h. A loopful (10 µl) of the enriched culture was then inoculated on MacConkey agar (HiMedia, Pvt. Ltd., India) supplemented with 1 mg l^−1^ cefotaxime (CTX) and incubated at 37 °C for 18 h. Colonies growing on this selective media were presumed to be potential β-lactamase-producing Gram-negative bacteria [34]. A single colony was randomly selected and subcultured onto Luria–Bertani (LB) agar supplemented with 1 mg l^−1^ CTX. When plates exhibited distinct colony morphologies, up to three colonies were subcultured separately to capture representative resistant phenotypes. While this approach may have limited the detection of within-sample diversity, it was designed to identify representative 3GC-resistant isolates across farms.

### Species identification of 3GC-resistant isolates

CTX-resistant Gram-negative bacterial isolates were initially characterized by biochemical tests and confirmed by PCR or Sanger sequencing. For instance, *Escherichia coli* was confirmed by PCR targeting the *yaiO* gene. However, because *yaiO* is also present in *Shigella sonnei* and *Shigella boydii* [35]*,* isolates positive for this gene were further screened for the invasion plasmid antigen H (*ipaH*) gene, which is found in all *Shigella* [36]. Meanwhile, other Gram-negative bacteria were identified by Sanger sequencing targeting the 16S rRNA gene*.*

Concisely, the Promega Wizard^®^ SV Gel and PCR Clean-Up System Purification Kit (Madison, WI, USA) was used to purify PCR products according to the manufacturer’s instructions. Next, cycle sequencing was performed with the BigDye Terminator v3.1 Kit (Applied Biosystems, Waltham, MA, USA), and the products were purified using Agencourt CleanSEQ magnetic beads and Terminator Removal (Beckman Coulter, MA, USA). Finally, the purified products were sequenced on the SeqStudio platform, and the resulting sequences were cleaned and assembled using SnapGene v7.2, followed by a blast search in the National Center for Biotechnology Information database.

### Antimicrobial susceptibility testing

Phenotypic AMR profiles were determined using the Kirby–Bauer disc diffusion method [37] on Mueller–Hinton (MH) agar (Oxoid, Germany). Gram-negative bacterial colonies were suspended in sterile saline to a turbidity equivalent to a 0.5 McFarland standard (~1.5×10^8^ c.f.u. ml^−1^) [38]. Sterile cotton swabs were used to evenly inoculate MH agar, after which antibiotic discs were placed, and the plates were incubated at 37 °C for 18 h.

Eight antibiotics were used, representing clinically important antimicrobial categories, and were selected based on local prescription patterns at the University Teaching Hospital, Lusaka [39]: imipenem (IPM, 10 µg), azithromycin (15 µg), gentamicin (10 µg), ciprofloxacin (5 µg), sulphamethoxazole/trimethoprim (25 µg), chloramphenicol (30 µg), tetracycline (30 µg) and ampicillin (10 µg). Results were interpreted according to Clinical and Laboratory Standards Institute (CLSI) guidelines [40]. The *E. coli* ATCC 25922 strain was used as a quality control.

### Characterization of carbapenem-resistant Enterobacterales

To quantify IPM resistance, the broth microdilution method was used [41]. To this end, Gram-negative bacterial isolates exhibiting resistance to IPM on the disc diffusion method were inoculated into LB broth supplemented with 1 mg l^−1^ IPM and incubated for 18 h at 37 °C with shaking at 170 r.p.m. The overnight cultures were then diluted 10^4^-fold [42] and inoculated, in triplicate, into 96-well microtitre plates containing twofold serial dilutions of IPM [43]. The 96-well plates were then incubated for 18 h at 37 °C with shaking at 170 r.p.m.

Bacterial growth was assessed by measuring ODs at 450 nm (OD_₄₅₀_) using a Bio-Rad iMark™ Microplate Reader (Bio-Rad Laboratories Inc., CA, USA). Wells with OD₄₅₀ ≥0.1 were considered to indicate positive growth. The MIC was defined as the lowest antibiotic concentration resulting in an OD_₄₅₀_ <0.1. IPM MICs were interpreted according to CLSI breakpoints [40]. The *E. coli* ATCC 25922 strain was used for quality control.

### Characterization of β-lactamase-producing Enterobacterales

PCR was conducted to screen for previously reported β-lactamase-encoding (*bla*) genes: *bla*_CTX-M_, *bla*_TEM_, *bla*_OXA-1_ and *bla*_SHV_. The primers used in this study were validated in previous reports and are shown in Table 1 [35,44,47]. Among the five known *bla*_CTX-M_ groups (1, 2, 8, 9 and 25), only groups 1, 2 and 9 were targeted, based on prior studies in Zambia, where only groups 1 and 2 have been reported [41,48]. Therefore, the primers CTX-MA1/CTX-MA2 [44], specific for *bla*_CTX-M_ groups 1, 2 and 9, were used in this study. Additionally, universal primers were used to independently detect *bla*_TEM_ and *bla*_SHV_, and specific primers were used for *bla*_OXA-1_.

**Table 1.** Primers used in this study

Primer name	Sequence (5′–3′)	Product size (bp)	Reference
CTX-MA1	^*^SCSATGTGCAG^†^YACCAGTAA	544	[44]
CTX-MA2	CCGC^‡^RATATGRTTGGTGGT		
TEM-F	GTATCCGCTCATGAGACAATA	717	[45]
TEM-R	AGAAGTGGTCCTGCAACTTT		
OXA-1-F	GGCACCAGATTCAACTTTCAAG	564	[46]
OXA-1-R	GACCCCAAGTTTCCTGTAAGTG		
SHV-F	AGGATTGACTGCCTTTTTG	392	[47]
SHV-R	ATTTGCTGATTTCGCTCG		
yaiO-F	TGATTTCCGTGCGTCTGAATG	115	[35]
yaiO-R	ATGCTGCCGTAGCGTGTTTC		
NDM-F	TTGGCCTTGCTGTCCTTG	82	[49]
NDM-R	ACACCAGTGACAATATCACCG		

*S = G or C.

†Y = C or T.

‡R = A or T.

CTX-MA1/2, Primer pair targeting blaCTX-M; NDM-F/R, Primer pair targeting blaNDM; OXA-1-F/R, Primer pair targeting blaOXA-1; SHV-F/R, Primer pair targeting blaSHV; TEM-F/R, Primer pair targeting blaTEM; yaiO-F/R, Primer pair targeting yaiO gene.

Genomic DNA was extracted from colonies of the 3GC-resistant isolates using DNAzol^®^ Genomic DNA Isolation Reagent (Molecular Research Centre, Inc., USA) following the manufacturer’s protocol. PCR was performed using Takara ExTaq HS (TaKaRa, Japan) with a total reaction volume of 25 µl, containing PCR buffer, deoxyribonucleoside triphosphate (dNTP) mixture, DNA template, primers, nuclease-free water and Takara Ex Taq DNA polymerase.

Thermocycling conditions for *bla*_CTX-M_, *bla*_TEM_ and *bla*_OXA-1_ were: initial denaturation at 94 °C for 1 min; 30 cycles of template denaturation at 94 °C for 30 s, primer annealing at 58 °C for 30 s and extension at 72 °C for 30 s; followed by a final extension at 72 °C for 5 min. For *bla*_SHV_, the same conditions were used except for an annealing temperature of 56 °C for 30 s.

PCR products were visualized under UV illumination after electrophoresis on a 1.5% agarose gel for 30 min. In addition, all CR-GNB identified by disc diffusion in this study were screened for the *bla*_NDM_ gene using previously described primers [49] listed in Table 1.

Hierarchical clustering was applied as an exploratory tool to visualize similarities in antimicrobial susceptibility testing (AST) results and *bla* genes, rather than to infer clonal relatedness.

## Results

### High prevalence of 3GC-resistant Gram-negative bacteria in poultry samples

A total of 45 pooled cloacal and 45 pooled meat samples were obtained from small-scale broiler farms in Lusaka Province and screened for the presence of 3GC-resistant Gram-negative bacteria. Multiple bacterial species were recovered from both cloacal and meat swabs, yielding a total of 83 isolates (42 cloacal and 41 meat). Among these, 57 were *E. coli* (i.e. positive for *yaiO* and negative for *ipaH* by PCR), while 26 were non-*E. coli* bacteria. *E. coli* was significantly more abundant in cloacal samples (*n*=38) than in meat (*n*=19) swabs (*χ²*, *P*=0.0000824) and exhibited a combined MDR rate of 89.5% (51/57) (Table 2).

**Table 2.** Prevalence of CTX-resistant Gram-negative bacteria (*n*=83)

Species	CTX resistance per source		Overall CTX resistance, *n* (%)	MDR frequency	
	Cloacal swab	Meat swab		*n*	%
**Enterobacterales**					
*E. coli*	38	19	57 (68.7)	51	89.5
*K. pneumoniae*	1	5	6 (7.2)	6	100
*Enterobacter* spp.	1	5	6 (7.2)	4	66.7
*C*. *freundii*	1	1	2 (2.4)	2	100
**Non-Enterobacterales**					
*P. aeruginosa*	1	3	4 (4.8)	3	75
Other *Pseudomonas* spp.	0	4	4 (4.8)	2	50
*A. baumannii*	0	3	3 (3.6)	1	33.3
*A*. *caviae*	0	1	1 (1.2)	1	100

**Fig. 1.**
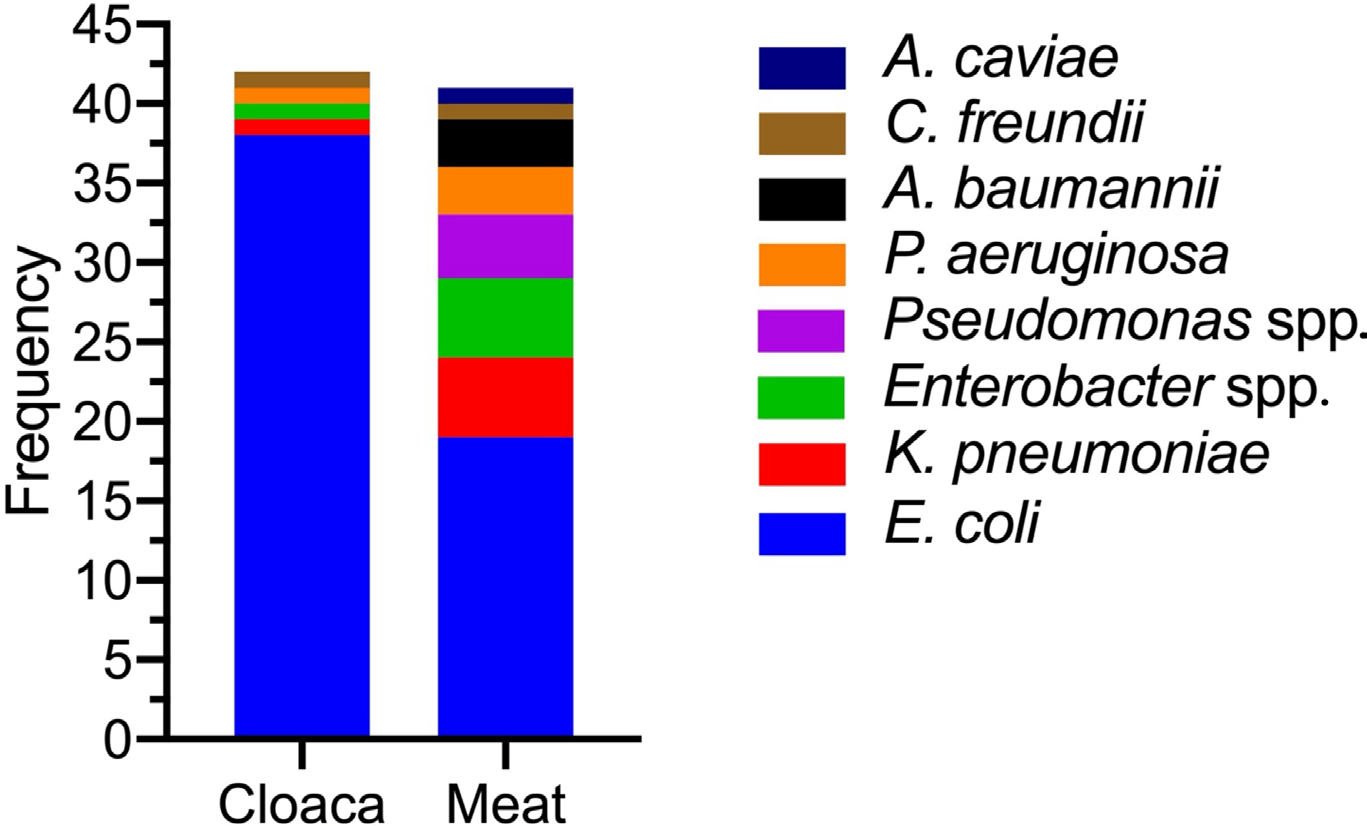
Distribution of CTX-resistant Gram-negative bacteria according to sample source. The sample sources were almost equally colonized, although swabs from meat showed greater species diversity. *A. caviae*, *C. freundii* and *Pseudomonas* species (excluding *P. aeruginosa*) were restricted to meat swabs.

In contrast, 22 out of 26 (84.6%) non-*E. coli* bacteria were isolated from meat swabs (84.6%), including Enterobacterales such as *Klebsiella pneumoniae* (*n=*5), *Enterobacter* species (*n=*5) and *Citrobacter freundii* (*n=*1). Non-Enterobacterales included *Pseudomonas aeruginosa* (*n=*3), other *Pseudomonas* spp. (*n=*4), *Acinetobacter baumannii* (*n=*3) and *Aeromonas caviae* (*n=*1). Notably, *Pseudomonas* spp.*, A. baumannii* and *A. caviae* were absent from cloacal swabs, suggesting contamination from an external source (Table 2, Fig. 1).

**Fig. 2.**
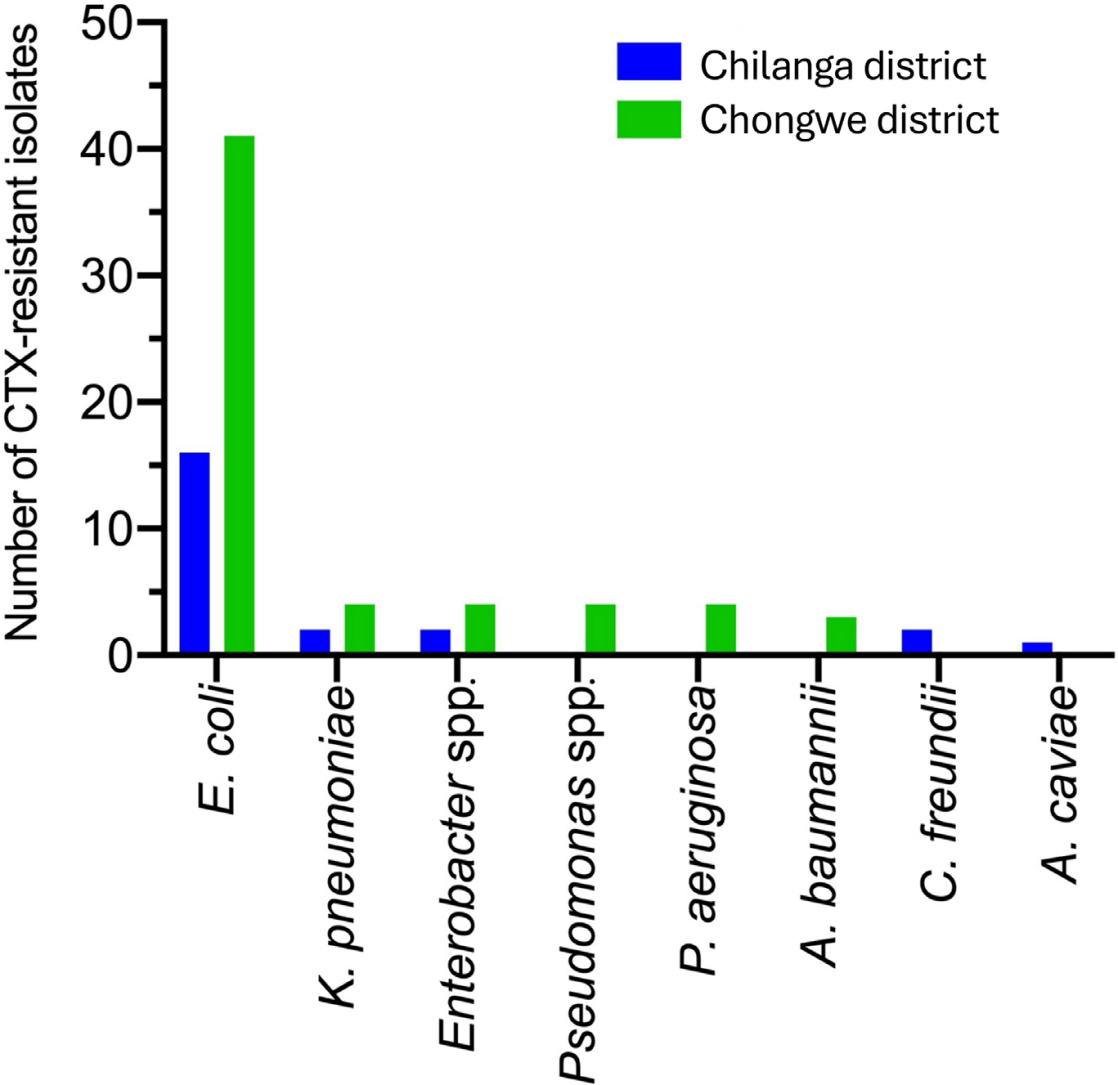
CTX-resistant Gram-negative bacteria isolated according to study district. The proportional isolation rates for *E. coli*, *K. pneumoniae* and *Enterobacter* species were similar between the two districts. *Pseudomonas* species were restricted to Chongwe district, while *C. freundii* and *A. caviae* were only isolated from Chilanga district samples.

Of the 83 3GC-resistant isolates, 60 originated from the 35 farms in Chongwe district and 23 from the ten farms in Chilanga district. *E. coli, K. pneumoniae* and *Enterobacter* spp. were detected in both districts. In contrast, *Pseudomonas* spp. and *A. baumannii* were found exclusively in Chongwe, and *C. freundii* and *A. caviae* were unique to Chilanga (Fig. 2).

### High rates of MDR among Enterobacterales

**Fig. 3.**
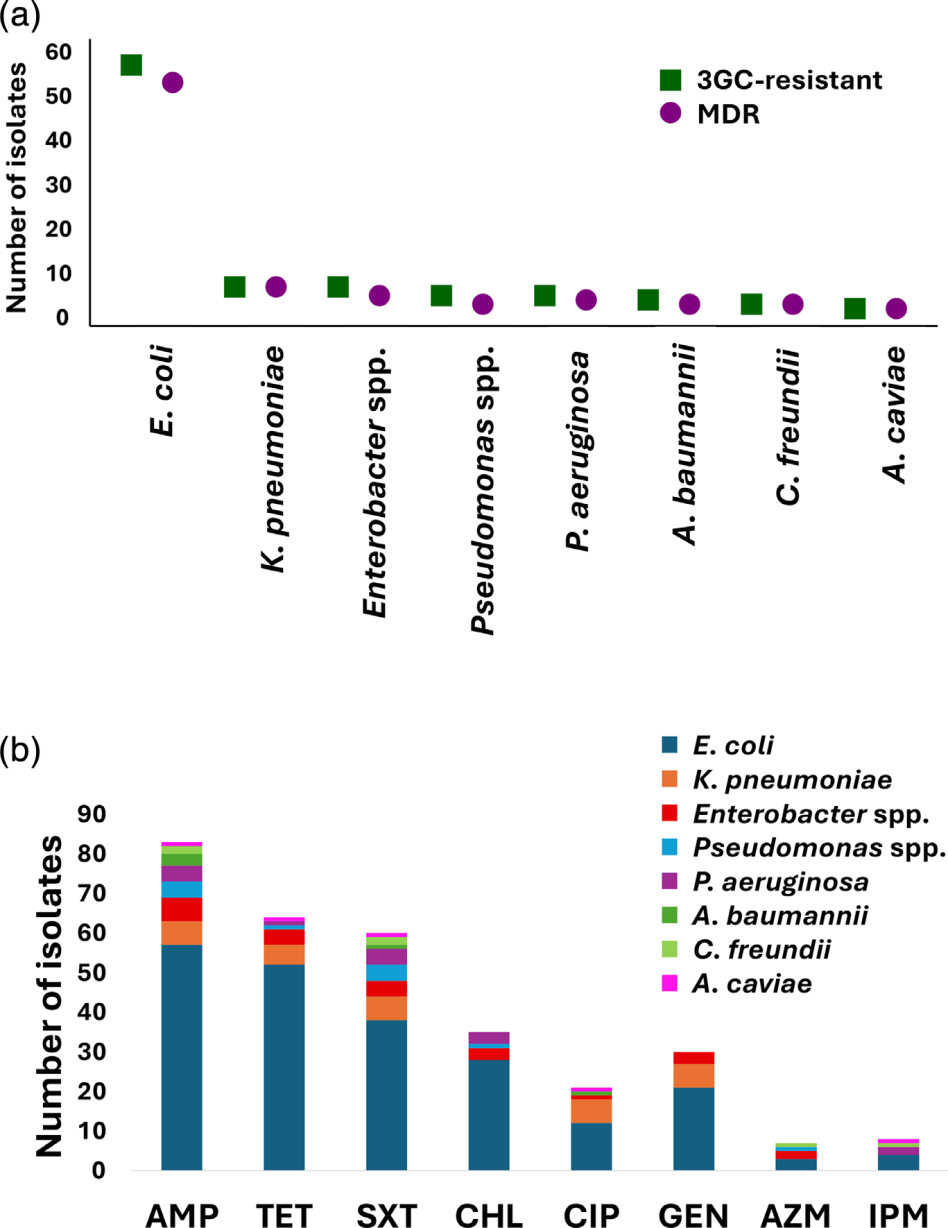
Multidrug-resistant phenotype among 3GC-resistant isolates. (a) Comparison of the number of 3GC-resistant isolates and those possessing MDR. (b) Number of isolates resistant to each antibiotic in different classes. All bacterial species were resistant to AMP, while only seven were resistant to AZM, and, notably, ten isolates were resistant to IPM. AMP, ampicillin; AZM, azithromycin; CHL, chloramphenicol; CIP, ciprofloxacin; GEN, gentamicin; SXT, sulphamethoxazole and trimethoprim; TET, tetracycline.

ASTs of all 83 3GC-resistant isolates against eight classes of antibiotics revealed that 70 (84.3%) displayed multidrug-resistant phenotypes. For the Enterobacterales, MDR was detected in *E. coli* (51/57, 89.5%), *K. pneumoniae* (6/6, 100%), *C. freundii* (2/2, 100%) and *Enterobacter* spp. (4/6, 66.7%). For non-Enterobacterales, MDR was detected in *A. baumannii* (1/3, 33.3%), *P. aeruginosa* (3/4, 75%), other *Pseudomonas* spp. (2/4, 50%) and *A. caviae* (1/1, 100%) (Fig. 3a, Table 2).

Resistance to ampicillin was universal (83/83, 100%), while high resistance levels were also observed for tetracycline (64/83, 77.1%) and co-trimoxazole (60/83, 72.3%). Fewer isolates were resistant to chloramphenicol (35/83, 42.2%), ciprofloxacin (21/83, 25.3%), gentamicin (30/83, 36.1%) and azithromycin (7/83, 8.4%) (Table 3). Following Ourghanlian *et al*. [50], isolates categorized as ‘intermediate’ (I) were reclassified as ‘susceptible, increased exposure’ and were thus considered ‘susceptible’ in the context of this study.

**Table 3.** AST by bacterial species

Antibiotics species	AMP		TET		SXT		CHL		CIP		GEN		AZM		IPM	
	S*	R†	S	R	S	R	S	R	S	R	S	R	S	R	S	R
**Enterobacterales**																
*E. coli* (*n*=57)	0	57	5	52	19	38	29	28	45	12	36	21	54	3	53	4
*K. pneumoniae* (*n*=6)	0	6	1	5	0	6	6	0	0	6	0	6	6	0	6	0
*Enterobacter* spp. (*n*=6)	0	6	2	4	2	4	3	3	5	1	3	3	4	2	6	0
*C. freundii* (*n*=2)	0	2	2	0	0	2	2	0	2	0	2	0	1	1	1	1
**Non-Enterobacterales**																
*Pseudomonas* spp. (*n*=4)	0	4	3	1	0	4	3	1	4	0	4	0	3	1	4	0
*P. aeruginosa* (*n*=4)	0	4	3	1	0	4	1	3	4	0	4	0	4	0	2	2
*A. baumannii* (*n*=3)	0	3	3	0	2	1	3	0	2	1	3	0	3	0	3	0
*A. caviae* (*n*=1)	0	1	0	1	0	1	1	0	0	1	1	0	1	0	0	1
**Overall totals (*n*=83**)	**0**	**81**	**19**	**64**	**23**	**60**	**48**	**35**	**62**	**21**	**53**	**30**	**76**	**7**	**73**	**10**

*Susceptible.

†Resistant.

AMP, ampicillin; AZM, azithromycin; CHL, chloramphenicol; CIP, ciprofloxacin; GEN, gentamicin; SXT, sulphamethoxazole and trimethoprim; TET, tetracycline.

Remarkably, ten out of 83 (12%) isolates in this study were resistant to IPM, a last-resort carbapenem for treating severe Gram-negative infections [51,52] (Fig. 3b). These isolates, identified via disc diffusion, retained resistance in broth microdilution assays, with IPM MICs ranging from 4 to 32 mg l^−1^. The highest MIC (32 mg l^−1^) was observed in *A. caviae*, followed by *P. aeruginosa* (IPM MIC=8 mg l^−1^, *n*=2), *C. freundii* (4 mg l^−1^, *n*=2) and *E. coli* (4 mg l^−1^, *n*=5) (Fig. S1, available in the online Supplementary Material).

### Distribution of *bla* genes among 3GC-resistant isolates

**Fig. 4.**
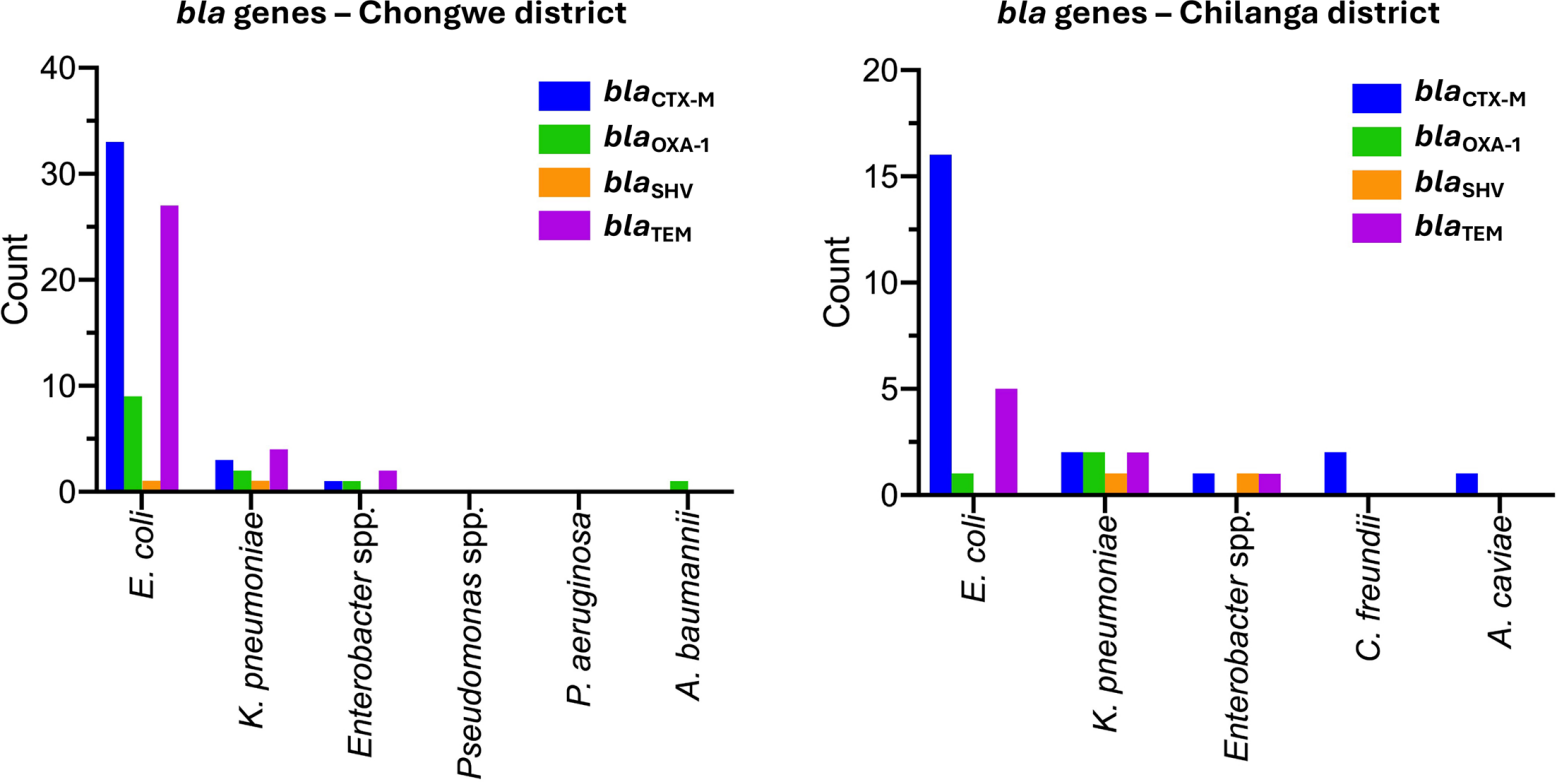
*bla* genes by district of isolate origin. There was no difference in the proportions of *bla* genes between Chongwe and Chilanga districts.

To determine the genotypic basis of resistance, PCR targeting *bla*_CTX-M_, *bla*_TEM_, *bla*_OXA-1_ and *bla*_SHV_ was performed. Overall, 80.7% (67/83) of the isolates harboured at least one *bla* gene, with *bla*_CTX-M_ being the most prevalent (66.2%), followed by *bla*_TEM_ (49.4%), *bla*_OXA-1_ (19.3%) and *bla*_SHV_ (4.8%). The proportions of *bla* genes did not differ significantly between Chongwe and Chilanga districts (Prop test, *P*>0.05) (Fig. 4).

**Fig. 5.**
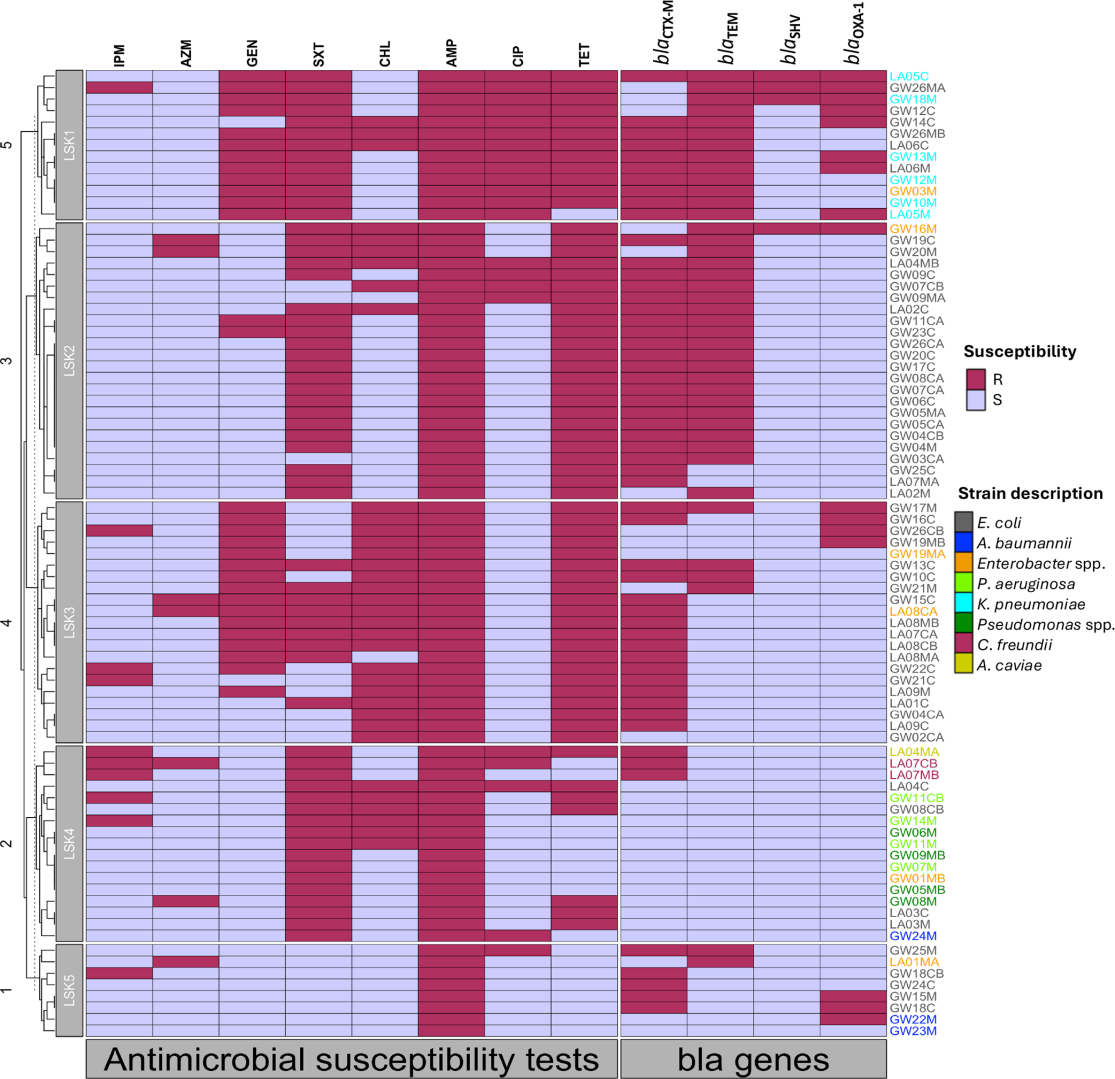
Dendrogram based on AST profiles and selected *bla* genes. Five clades (LSK1–LSK5) were identified, each containing isolates from both Chongwe and Chilanga. These clusters reflect shared resistance profiles rather than confirmed clonal relationships. LSK1 and LSK3 showed the highest aminoglycoside resistance, LSK4 the greatest species diversity, and LSK5 the highest susceptibility. LSK2 was dominated by *E. coli* co-harbouring *bla*_CTX-M_ and *bla*_TEM_. Maroon and purple rectangles indicate the presence or absence of *bla* genes, respectively; The prefixes LA and GW denote isolates from Chilanga and Chongwe, respectively. AMP, ampicillin; AZM, azithromycin; CHL, chloramphenicol; CIP, ciprofloxacin; GEN, gentamicin; R, resistant; S, susceptible; SXT, sulphamethoxazole and trimethoprim; TET, tetracycline.

At the species level, 96.5% (55/57) of *E. coli* isolates carried at least one *bla* gene, with 46.7% (28/60) harbouring both *bla*_CTX-M_ and *bla*_TEM_. Other *bla*-positive isolates included, for Enterobacterales, all six (6/6, 100%) *K. pneumoniae*, four (4/6, 66.7%) *Enterobacter* spp. and two (2/2, 100%) *C. freundii*. Among non-Enterobacterales, *bla* genes were detected in one (1/3, 33.3%) *A. baumannii* and one (1/1, 100%) *A. caviae*. Neither *P. aeruginosa* nor other *Pseudomonas* spp*.* possessed any of the screened *bla* genes (Figs 4 and 5). None of the 3GC-resistant isolates in this study tested positive for *bla*_NDM_.

### Hierarchical clustering of isolates by AST and selected *bla* genes

A dendrogram based on AST profiles and a limited panel of *bla* genes revealed five major clusters, here designated as LSK1–LSK5 (i.e Lusaka 1-5) (Fig. 5). Each clade contained isolates from both Chongwe and Chilanga, indicating no clear district-level separation based on the available phenotypic and genotypic data. Cluster LSK1 was dominated by *K. pneumoniae* and *E. coli* isolates, all of which exhibited fluoroquinolone resistance; 92.3% (12/13) were also aminoglycoside resistant. Furthermore, co-existence of *bla*_CTX-M_ and *bla*_TEM_ was observed in 76.9% (10/13) (Table S2) of LSK1 isolates. The two major clusters (LSK2 and LSK3) were dominated by *E. coli* isolates, with LSK3 showing a notable association with chloramphenicol resistance. LSK4 was dominated by *Pseudomonas* spp. lacking *bla* genes, while LSK5 comprised multiple species, including *Enterobacter* spp. and *A. baumannii*, and displayed more heterogeneous but generally lower resistance levels.

## Discussion

In this study, pooled cloacal and chicken meat samples from Chongwe and Chilanga districts of Lusaka Province, Zambia, were screened for carbapenem- and 3GC-resistant Gram-negative bacteria. Seven Gram-negative species were identified, with a high prevalence of 3GC resistance associated with MDR. Notably, 12% of isolates exhibited resistance to IPM, the antibiotic of last resort, signalling the emergence of carbapenem resistance, even though its detection was limited to our study sites. Genotypic characterization revealed a predominance of *bla*_CTX-M_ and *bla*_TEM_, which co-existed in nearly half of the *E. coli* strains, while *bla*_SHV_ and *bla*_OXA-1_ were less frequent. None of the isolates possessed the *bla*_NDM_ gene. Finally, a dendrogram based on ASTs and a limited panel of *bla* genes revealed clusters with isolates from both study districts, suggesting shared resistance characteristics across study sites.

The high prevalence of MDR among isolates in this study is concerning, as chickens are a primary and relatively inexpensive source of animal protein in Zambia and are commonly raised in proximity to humans. This proximity increases the risk of bidirectional transmission of drug-resistant bacteria between chickens and humans [48,53]. The observed results can be attributed to the high usage of antimicrobials in the poultry sector in Zambia [10]. The 2023 livestock survey reported that the main challenges for broiler chicken-raising households in Zambia include disease (41.6%), inadequate feed (9.4%) and limited breeding technology (4.1%) [30]. Such constraints compel farmers to resort to antibiotic use, particularly penicillins, tetracyclines and sulphonamides [10], which are readily available over the counter [54]. This could explain the high levels of ampicillin, tetracycline and co-trimoxazole resistance observed in this study. The widespread resistance across multiple antibiotic classes highlights the selective pressure imposed by indiscriminate antibiotic use in poultry production in Zambia, underscoring the urgent need for antimicrobial stewardship and continuous AMR surveillance. Similarly, a study assessing the prevalence and factors associated with multidrug-resistant *E. coli* carriage on chicken farms in Uganda also showed high resistance to ampicillin, tetracycline and co-trimoxazole, highlighting that multidrug-resistant *E. coli* carriage is highly prevalent on chicken farms [55]. Another report on AMR profiles of *E. coli* isolated from chickens in Kenya also showed the highest resistance to these three antibiotics [56].

While 3GCs are not routinely used in Zambian poultry production, resistance to this drug class has become common, likely due to the co-selection of *bla* genes on plasmids harbouring other AMR genes [20]. For instance, using tetracyclines in poultry would indirectly select for ESBL-producing bacteria if the *bla* and *tet* genes co-exist on the same plasmid. This observation aligns with previous reports of high ESBL prevalence in poultry isolates from Zambia [20]. It is also consistent with reports from other African countries, including Senegal, where *E. coli* isolates from healthy chicken farms showed 3GC resistance [57], and Nigeria, where ESBL-producing *E. coli* were detected in poultry and farm environments, reflecting widespread 3GC resistance in poultry production [58,59].

In Zambia, earlier studies reported susceptible phenotypes against carbapenems [9,60, 61]; however, our results revealed a 12% prevalence of carbapenem resistance. This discrepancy could be attributed to adaptive resistance, carbapenemases, overexpression of *ampC* genes or penicillin-binding protein (PBP) modification [62,66]. However, the exact resistance mechanism remains uncertain; thus, whole-genome sequencing (WGS) is essential to identify the underlying resistance mechanisms. Understanding these mechanisms has critical implications for control strategies. For instance, carbapenem resistance due to a modified PBP typically spreads clonally [67], whereas carbapenemase-associated resistance can disseminate horizontally across strains and species through mobile genetic elements [68]. Controlling clonal spread in clinical or animal settings often involves identifying infection sources and enforcing strict biosecurity and sanitation measures, while managing horizontal gene transfer requires more complex approaches such as conjugation inhibitors [69], Clustered Regularly Interspaced Short Palindromic Repeats - CRISPR-associated (CRISPR-Cas) systems [70], phage therapy [71] and anti-plasmid compounds [72]. Regardless of the transmission dynamics, the detection of carbapenem-resistant isolates in this study is alarming, as zoonotic transmission could have profound clinical implications. This is especially critical in Zambia, where colistin, a last-resort antibiotic against multidrug-resistant Gram-negative infections, is unavailable for clinical use, and most hospitals lack the capacity to monitor its nephrotoxic effects [44].

Evidence of CR-GNB in poultry is still relatively scarce in Africa, but reports are emerging. For example, one study in Egypt documented carbapenem-resistant *K. pneumoniae* isolates from broiler chickens, their environment and farm workers, all carrying carbapenemase-encoding genes [73]. Elsewhere, CR-GNB have been detected in hospital wastewater, representing a significant environmental health risk [74]. Such effluents frequently contain carbapenemase-encoding genes and other AMR determinants [75]. In Zambia, carbapenems are more commonly used in high-level referral hospitals in Lusaka. While data from this study cannot directly confirm transmission pathways, environmental contamination is a plausible source of exposure to resistant bacteria for livestock [76], thereby facilitating the spread of resistant organisms through the environment–animal–human interface [77].

In our study, most of the carbapenem-resistant isolates had IPM MICs near the breakpoint, but two exhibited high resistance (IPM MICs of 32 and 8 mg l^−1^), which may indicate carbapenemase production. As these isolates tested negative for *bla*_NDM_, carbapenemase production cannot be excluded. The isolates should be further screened for other carbapenemase-encoding genes, such as *bla*_KPC_, *bla*_OXA-48_, *bla*_VIM_ and *bla*_IMP_, since current screening does not allow comprehensive characterization of carbapenem resistance mechanisms. WGS would enable detection of a broader range of carbapenemase genes, determining whether these genes are plasmid-borne or chromosomal, and help identify potential transmission pathways. While carbapenem resistance in Enterobacterales is often attributed to carbapenemase enzymes with high transmissibility, it should be noted that carbapenem resistance in *Pseudomonas* spp. and *Acinetobacter* spp. is driven by different mechanisms, such as porin loss, efflux pump overexpression and intrinsic β-lactamases [78]. Consequently, phenotypic interpretations and clinical implications differ between these groups.

The predominance of *bla*_CTX-M_ and *bla*_TEM_ observed here is consistent with prior reports from Zambian poultry and clinical isolates [9,41]. Similarly, a review on ESBLs in poultry in Africa reported that in 30.3% of studies (ten out of 33), the co-existence of ESBL genes was observed, and in nine of those studies, the genes *bla*_CTXM_ and *bla*_TEM_ were detected together within the same ESBL-producing *E. coli* strains [79]. The frequent co-existence of these genes suggests possible localization on the same plasmid [25,80]. Therefore, detecting these genes at the point of care could help medical professionals make well-informed decisions about which antibiotics to administer. However, molecular diagnostics remain limited in Zambia, with most programmes relying solely on phenotypic ASTs. Data from this and previous studies could therefore inform the design of rapid PCR-based diagnostic tools targeting prevalent *bla* genes and bacterial species, enhancing national AMR surveillance.

Cluster analysis, based on phenotypic and genotypic data, revealed that *E. coli* dominated two major clusters (LSK2 and LSK3), *K. pneumoniae* dominated LSK1, and all *Pseudomonas* spp. grouped in LSK4. Isolates from both study districts were represented in each cluster, suggesting shared resistance profiles across sites. However, inference beyond the study districts, including across Lusaka Province, should be made with caution, as phenotypic similarity combined with a limited gene panel cannot reliably distinguish clonal dissemination from unrelated strains exhibiting convergent resistance patterns. Interestingly, *Pseudomonas* spp. lacked all tested *bla* genes but exhibited MDR, which may be attributed to intrinsic resistance mechanisms such as efflux pump overexpression and low outer membrane permeability [81], or the presence of other AMR genes, including those encoding AmpC β-lactamases. To confirm these hypotheses, WGS and a detailed comparison of sequence types, O-antigen : H-antigen (OH) serotypes, plasmid replicons and AMR genes are needed.

While this study provides valuable insights, it has a few notable limitations. First, only four AMR genes were evaluated, and WGS was not performed, limiting insight into strain-level diversity and resistance determinants. Therefore, future studies should conduct WGS to explore all known AMR genes and mutations. Despite this limitation, the results of this study are consistent with those of previous WGS-based studies [41,48]. Second, pooling samples meant that results reflected dominant strains in the population, potentially underestimating microbial diversity. Individual-level sampling could reveal additional diversity. Third, the study was limited to two districts, which may limit its generalizability to other regions in Zambia. Finally, this study was based only on isolates collected from poultry; however, understanding the complex transmission dynamics of MDR requires a multipronged approach that considers various potential hosts and habitats. Therefore, we recommend that future studies adopt a One Health approach, incorporating clinical, animal and environmental samples to provide a comprehensive understanding of AMR transmission across interconnected ecosystems.

## Conclusion

This study identified a high prevalence of 3GC- and CR-GNB with MDR profiles in poultry from two districts of Lusaka Province, Zambia, highlighting the growing public health threat. The widespread resistance to commonly used antibiotics indicates ongoing misuse and gaps in antimicrobial stewardship within the poultry sector. Notably, 12% of the isolates were resistant to IPM, the antibiotic of last resort, representing a relatively high prevalence and emphasizing the need for continuous surveillance. These findings underscore the urgency of strengthening antimicrobial stewardship, regulating antibiotic use in food-producing animals and integrating genomic surveillance under a One Health framework to mitigate the emergence and dissemination of carbapenem- and multidrug-resistant bacteria across human, animal and environmental sectors.

## Supplementary material

10.1099/acmi.0.001108.v4 Uncited Supplementary Material 1.

10.1099/acmi.0.001108.v4 Uncited Supplementary Material 2.

## Abbreviations

AMP: ampicillin
AMR: antimicrobial resistance
AST: antimicrobial susceptibility testing
AZM: azithromycin
blaCTX-M: cefotaximase-Munich-encoding gene
blaOXA-1: oxacillinase-1-encoding gene
blaSHV: sulfydryl variable-encoding gene
blaTEM: Temoneira type-encoding gene
CHL: chloramphenicol
CIP: ciprofloxacin
CLSI: Clinical and Laboratory Standards Institute
CR-GNB: carbapenem-resistant Gram-negative bacteria
CTX: cefotaxime
CTX-M: cefotaximase-Munich
ESBL: extended-spectrum β-lactamase
3GCs: third-generation cephalosporins
GEN: gentamicin
ipaH: invasion plasmid antigen H
IPM: imipenem
LB: Luria–Bertani
MDR: multidrug resistance
MH: Mueller–Hinton
OXA-1: oxacillinase-1
PBP: penicillin-binding protein
SHV: sulphydryl variable
SXT: sulphamethoxazole and trimethoprim
TEM: temoneria
TET: tetracycline
WGS: whole-genome sequencing
