# Supplementary material for: Detection of carbapenem resistance among third-generation cephalosporin-resistant Enterobacterales from small-scale poultry farms in peri-urban Lusaka, Zambia
Source: Access Microbiol. 2026 Jan 28;8(1):001108.v4. doi: 10.1099/acmi.0.001108.v4 (PMC12852006; doi:10.1099/acmi.0.001108.v4)
Supplement: Uncited Supplementary Material 1. [file acmi-8-01108-s001.pdf]

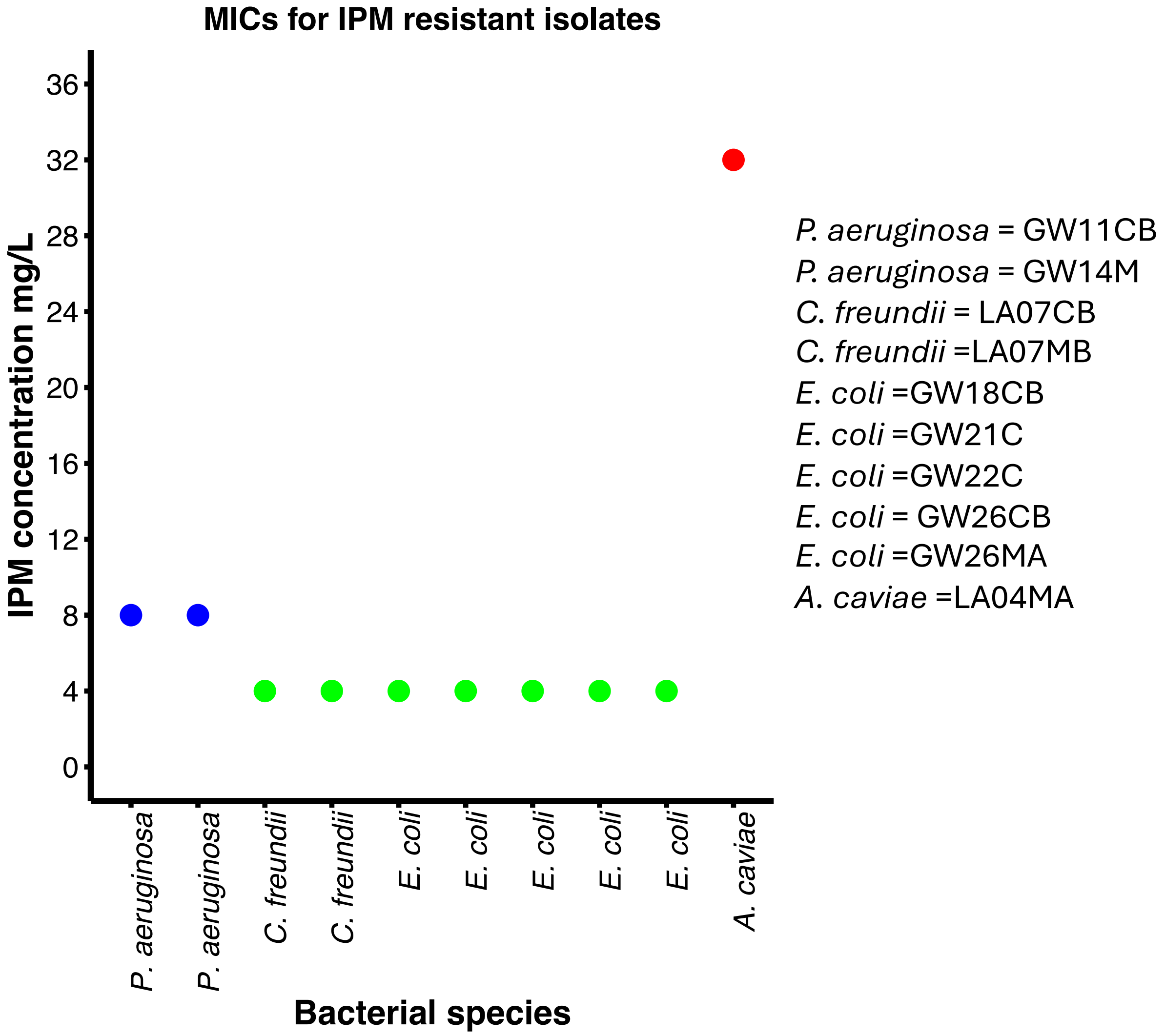

Figure S1: MICs for imipenem resistant isolates. *A. caviae* had the highest MIC at 32 mg/L, followed by the two *P. aeruginosa* isolates at 8 mg/L. *C. freundii* and *E. coli* had MICs of 4 mg/L.

Table S2: Antibiotic resistance and *bla* gene distribution

**Clade**	**No. of isolates**	**Antibiotic resistance, n (%)**								***bla* genes present n (%)**			
	(n)	IPM	AZM	GEN	SXT	CHL	AMP	CIP	TET	bla_{CTX-M}	bla_{TEM}	bla_{OXA-1}	bla_{SHV}
LSK1	13	1 (7.7%)	0 (0%)	12 (92.3%)	13 (100%)	3 (23.1%)	13 (100%)	13 (100%)	12 (92.3%)	10 (76.9%)	13 (100%)	3 (23.1%)	8 (61.5%)
LSK2	24	0 (0%)	2 (8.3%)	2 (8.3%)	21 (87.5%)	6 (25%)	24 (100%)	4 (%) 16.7%	21 (87.5%)	21 (87.5%)	22 (91.7%)	1 (4.2%)	1 (4.2%)
LSK3	21	3 (14.3%)	2 (9.5%)	16 (76.2%)	9 (42.9%)	20 (95.2%)	21 (100%)	0 (0%)	21 (100%)	16 (76.2%)	4 (19%)	0 (0%)	4 (19%)
LSK4	17	5 (29.4%)	2 (11.8%)	0 (0%)	17 (100%)	6 (35.3%)	17 (100%)	4 (23.5%)	7 (41.2%)	3 (17.6%)	0 (0%)	0 (0%)	0 (0%)
LSK5	8	1 (12.5%)	1 (12.5%)	0 (0%)	0 (0%)	0 (0%)	8 (100%)	1 (12.5%)	0 (0%)	5 (%)	2 (25%)	0 (0%)	3 (37.5%)
